# Synaptic Plasticity Dynamics for Deep Continuous Local Learning (DECOLLE)

**DOI:** 10.3389/fnins.2020.00424

**Published:** 2020-05-12

**Authors:** Jacques Kaiser, Hesham Mostafa, Emre Neftci

**Affiliations:** ^1^FZI Research Center for Information Technology, Karlsruhe, Germany; ^2^Department of Bioengineering, University of California, San Diego, La Jolla, CA, United States; ^3^Department of Cognitive Sciences, University of California, Irvine, Irvine, CA, United States; ^4^Department of Computer Science, University of California, Irvine, Irvine, CA, United States

**Keywords:** spiking neural network, embedded learning, neuromorphic hardware, surrogate gradient algorithm, backpropagataon

## Abstract

A growing body of work underlines striking similarities between biological neural networks and recurrent, binary neural networks. A relatively smaller body of work, however, addresses the similarities between learning dynamics employed in deep artificial neural networks and synaptic plasticity in spiking neural networks. The challenge preventing this is largely caused by the discrepancy between the dynamical properties of synaptic plasticity and the requirements for gradient backpropagation. Learning algorithms that approximate gradient backpropagation using local error functions can overcome this challenge. Here, we introduce Deep Continuous Local Learning (DECOLLE), a spiking neural network equipped with local error functions for online learning with no memory overhead for computing gradients. DECOLLE is capable of learning deep spatio temporal representations from spikes relying solely on local information, making it compatible with neurobiology and neuromorphic hardware. Synaptic plasticity rules are derived systematically from user-defined cost functions and neural dynamics by leveraging existing autodifferentiation methods of machine learning frameworks. We benchmark our approach on the event-based neuromorphic dataset N-MNIST and DvsGesture, on which DECOLLE performs comparably to the state-of-the-art. DECOLLE networks provide continuously learning machines that are relevant to biology and supportive of event-based, low-power computer vision architectures matching the accuracies of conventional computers on tasks where temporal precision and speed are essential.

## 1. Introduction

Understanding how the plasticity dynamics in multilayer biological neural networks are organized for efficient data-driven learning is a long-standing question in computational neurosciences (Sussillo and Abbott, 2009; Clopath et al., 2010; Zenke and Ganguli, 2017). The generally unmatched success of deep learning algorithms in a wide variety of data-driven tasks prompts the question of whether the ingredients of their success are compatible with their biological counterparts, namely SNNs. Biological neural networks distinguish themselves from ANNs by their continuous-time dynamics, the locality of their operations (Baldi et al., 2017), and their spike(event)-based communication. Taking these properties into account in a neural network is challenging, as the spiking nature of the neurons' nonlinearity makes it non-differentiable, the continuous-time dynamics raise a temporal credit assignment problem and the assumption of computations being local to the neuron disqualifies the use of Back-Propagation-Through-Time (BPTT).

**Figure 1:**
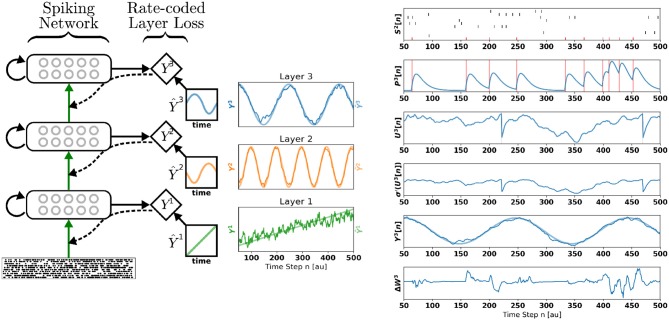
Deep Continuous Local Learning (DECOLLE). **(Left)** Each layer consists of spiking neurons with continuous dynamics. Each layer feeds into a local readout units through fixed, random connections (diamond-shaped, *y*). The local layer is trained such that the readout at each layer produce auxiliary targets Ŷ. Errors are propagated through the random connections to train weights coming into the spiking layer, but no further (curvy, dashed line). To simplify the learning rule and enable linear scaling of the computations, the cost function is a function of the states in the same time step. The state of the spiking neurons (membrane potential, synaptic states, refractory state) is carried forward in time. Consequently, even in the absence of recurrent connections, the neurons are stateful in the sense of recurrent neural networks. **(Right)** Snapshot of the neural states illustrating the DECOLLE learning rule in the top layer. In this example, the network is trained to produce three time-varying pseudo-targets Ŷ^1^, Ŷ^2^, Ŷ^3^.

In this article, we describe DECOLLE, a Spiking Neural Network (SNN) model with plasticity dynamics that solves the three problems above, and that performs at proficiencies comparable to that of multilayer neural networks. DECOLLE uses layerwise local readouts (Mostafa et al., 2017), which enables gradients to be computed locally (Figure 1). To tackle the temporal dynamics of the neurons, we use a recently established equivalence between SNNs and recurrent ANNs (Neftci et al., 2019). This equivalence rests on a computational graph of the SNN, which can be implemented with standard machine learning frameworks as a recurrent neural network. Unlike BPTT and like Real-Time Recurrent Learning (RTRL) (Williams and Zipser, 1989), DECOLLE is formulated in a way that the information necessary to compute the gradient is propagated forward, making the plasticity rule temporally local. Existing rules of this sort require dedicated state variables for every synapse, thus scaling at least quadratically with the number of neurons (Williams and Zipser, 1989; Zenke and Ganguli, 2017). In contrast, DECOLLE scales linearly with the number of neurons. This is achieved using a spatially and temporally local cost function reminiscent of readout mechanisms used in liquid state machines (Maass et al., 2002), but where the readout is performed over a fixed random combination of the neuron outputs. Our approach can be viewed as a type of synthetic gradient, a technique used to decouple one or more layers from the rest of the network to prevent layerwise locking (Jaderberg et al., 2016). Although synthetic gradients usually involve an outer loop that is equivalent to a full Back-Propagation (BP) through the network, DECOLLE instead relies on the random initialization of the local readout and forgoes the outer loop.

Conveniently, DECOLLE can leverage existing autodifferentiation tools of modern machine learning frameworks. Its linear scalability enables the training of hundreds of thousands of spiking neurons on a single GPU, and continual learning on very fine time scales. We demonstrate our approach on the classification of gestures, the IBM DvsGesture dataset (Amir et al., 2017), recorded using an event-based neuromorphic sensor and report comparable performance to deep neural networks and even networks trained with BPTT.

### 1.1. Related Work

Previous work demonstrated learning in multiple layers of SNN using feedback alignment (Lillicrap et al., 2016; Neftci et al., 2017), performing at about 2% classification error on MNIST. However, those networks operated in the firing rate regime, by using either large populations or slow dynamics. In those works, training was not insensitive to the temporal dynamics of the neurons. The need for temporal dynamics are often obfuscated by the static nature of the benchmarked problems (e.g., MNIST), and a long readout interval that allows to ignore initial transients caused by the dynamics. In our previous work (Neftci et al., 2017), ignoring temporal dynamics raised a “loop duration” problem, i.e., that the errors are available only after they have propagated through the network. This introduces latency or requires additional buffers for storing intermediate neural states. In traditional deep learning, the loop duration manifests itself as “layerwise locking,” during which a layer's weights cannot be updated until a global cost function is evaluated (Jaderberg et al., 2016). This causes under utilization of the computing resources and a slowdown in learning. Besides the loop duration problem, multilayer networks trained with feedback alignment cannot reach the performances of gradient BP, especially with deeper networks (≥30% accuracy drop on ImageNet compared to backpropagation; Bartunov et al., 2018).

The complex dynamics of spiking neurons is an important feature that can be exploited for learning spatiotemporal patterns. In a single layer of neurons, this feature can be leveraged using gradient descent, since it is applicable to the subthreshold dynamics of leaky Integrate & Fire (I&F) neurons (Bohte et al., 2000; Gütig and Sompolinsky, 2006). Because the I&F neuron output is non-differentiable, however, the application of these approaches to multiple layers is not straightforward. To deal with this problem, SuperSpike uses a surrogate network with differentiable activation functions to compute an approximate gradient (Zenke and Ganguli, 2017). The authors show that this learning rule is equivalent to a forward-propagation of errors using synaptic traces, and is capable of learning in hidden layers of feedforward multilayer networks.

Because the traces need to be computed for every trainable parameter, Superspike scales temporally and spatially as *O*(*N*^2^), where *N* is the number of neurons. While the complex biochemical processes at the synapse could account for the quadratic scaling, it prevents an efficient implementation in available hardware. Like SuperSpike, DECOLLE uses surrogate gradients to perform weight updates, but as discussed later, the cost function is local in time and space, such that only one trace per input neuron is required. This enables the algorithm to scale linearly in space. Furthermore, in DECOLLE the computation of the gradients can reuse the variables computed for the forward dynamics, such that learning has no additional memory overhead.

DECOLLE has some resemblance with reservoir networks, which are neural networks with fixed internal connectivity and trainable readout functions (Jaeger, 2001; Maass et al., 2002; Eliasmith and Anderson, 2004; Sussillo and Abbott, 2009). The local readout in DECOLLE acts like a decoder layer in the flavor of the linear readouts in reservoir networks. In contrary to reservoir networks, DECOLLE learns the internal weights, but the readout weights are random and fixed. The training of the internal weights allows the network to learn representations that are easier to classify inputs for subsequent layers (Mostafa et al., 2017).

Spiking neural networks can be viewed as a subclass of binary, recurrent ANNs (Neftci et al., 2019). In the Artificial Neural Network (ANN) sense, they are recurrent even when all the connections are feed-forward because the neurons maintain a state that is propagated forward at every time step. Binary neural networks, where both activations and/or weights are binary were studied in deep learning as a way to decrease model complexity during inference (Courbariaux et al., 2016; Rastegari et al., 2016). BPTT for training SNNs was investigated in Bohte et al. (2000), Lee et al. (2016), Huh and Sejnowski (2017), Shrestha and Orchard (2018), and Bellec et al. (2018). BPTT-based approaches provide an unbiased estimation of the gradients but at a cost in memory, because the entire sequence and resulting activity states are stored to compute gradients. Although the truncation of the sequences (as in truncated BPTT) can mitigate this problem, it is not adequate when discretizing continuous-time networks, such as the SNN (Neftci et al., 2019) because the sequences can consists of hundreds of steps. This is because the time constants and simulation timestep in SNNs are such that the truncation window must be much larger. For SNN simulations with biological time constants, it is common to use simulation time steps Δ*t* ≤ 1ms. Smaller time steps capture non-linear dynamics more accurately and determine the temporal precision of all produced spike times. Assuming Δ*t* = 1ms (as used in this work), and if relevant interactions occur at one second, this implies that the truncation window must be at about 1,000 timesteps. This significantly increases the complexity of BPTT in SNNs. In practice, the size of SNN trainable by BPTT is severely limited by the available GPU memory (Shrestha and Orchard, 2018). As we explain later in this article, DECOLLE requires an order *T* less memory resources compared to BPTT, where *T* is the sequence length. Hence, DECOLLE networks are generally not memory-limited. Furthermore, DECOLLE can be formulated as a local, three-factor synaptic plasticity rule, and is thus amenable to implementation in dedicated, event-based (neuromorphic) hardware (Davies et al., 2018), and compatible with neurobiology.

Decoupled Neural Interfaces (DNI) were proposed to mitigate layerwise locking in training deep neural networks (Jaderberg et al., 2016). In DNIs, this decoupling is achieved using a synthetic gradient, a neural network that estimates the gradients for a portion of the network. In an inner loop, the network parameters are trained using the synthetic gradients, and in an outer loop, the synthetic gradient network parameters are trained using a full BP step. The gradient computed using local errors in DECOLLE described below can be viewed as a type of synthetic gradient, which ignores the outer loop to avoid a full BP step. Although ignoring the outer loop limits DECOLLE's adaptation of the features using errors from other layers, we find that the network performs at or above state-of-the-art accuracy on N-MNIST and DVS Gesture benchmark tasks.

A related method called E-prop was developed in parallel to DECOLLE (Bellec et al., 2019). The resulting learning rule in E-prop is of the same form as Superspike and DECOLLE. E-prop uses adaptive spiking Long Short Term Memory (LSTM) neurons to maintain a longer term memory. This generalization allows to solve tasks with long-term dependencies (similar to LSTMs) but requires maintaining one trace per synapse. These memory requirements quickly exceed the capabilities of modern GPUs, especially when applied to convolutional neural networks. Even in neuromorphic hardware, maintaining a synapse-specific trace can incur a prohibitive cost in area and power (Huayaney et al., 2016; Davies et al., 2018). In DECOLLE, we focus on networks which do not incur any memory overhead for training, allowing to tractably train large networks.

This work builds on a combination of how gradients are dynamically computed in SuperSpike and local errors. We show in the methods section that this combination considerably reduces the computational requirements compared to a computing a global loss.

## 2. Methods

### 2.1. Neuron and Synapse Model

The neuron and synapse models used in this work follow leaky, current-based I&F dynamics with a relative refractory mechanism. The dynamics of the membrane potential *U*_*i*_ of a neuron *i* is governed by the following differential equations:

(1) $$\begin{array}{l} \begin{array}{c} \;U_{i}\left(t\right)=V_{i}\left(t\right)-\rho R_{i}\left(t\right)+b_{i},\\ \tau_{mem}\frac{\text{d}}{\text{d}t}V_{i}\left(t\right)=-V_{i}\left(t\right)+I_{i}\left(t\right),\\ \;\tau_{ref}\frac{\text{d}}{\text{d}t}R_{i}\left(t\right)=-R_{i}\left(t\right)+S_{i}\left(t\right), \end{array} \end{array}$$

with *S*_*i*_(*t*) the binary value (0 or 1) representing whether neuron *i* spiked at time *t*. The separation of the membrane potential into two variables *U* and *V* is done here for implementations reasons only. Biologically, the two states can be interpreted as a special case of a two-compartment model, consisting of one dendritic (*V*) and one somatic (*U*) compartment (Gerstner et al., 2014, Chapter 6.4). The absence of dynamics for *U* can be interpreted as the special case when somatic capacitance is much smaller than the distal capacitance. A spike is emitted when the membrane potential reaches a threshold *S*_*i*_(*t*) = Θ(*U*_*i*_(*t*)), where Θ(*x*) = 0 if *x* < 0, otherwise 1 is the unit step function. The constant *b*_*i*_ represents the intrinsic excitability of the neuron. The refractory mechanism is captured with the dynamics of *R*_*i*_: the neuron inhibits itself after firing, by a constant weight ρ. In contrast to standard I&F refractory mechanisms, a strong enough input can still induce the neuron to fire immediately after a spike. The factors τ_*ref*_ and τ_*mem*_ are time constants of the membrane and refractory dynamics, respectively. *I*_*i*_ denotes the total synaptic current of neuron *i*, expressed as:

(2) $$\begin{array}{l} \begin{array}{c} \tau_{syn}\frac{\text{d}}{\text{d}t}I_{i}\left(t\right)=-I_{i}\left(t\right)+\underset{j\in \text{pre}}{\sum }W_{ij}S_{j}\left(t\right), \end{array} \end{array}$$

where *W*_*ij*_ is the synaptic weights between pre-synaptic neuron *j* and post-synaptic neuron *i*. Because *V*_*i*_ and *I*_*i*_ are linear with respect to the weights *W*_*ij*_, The dynamics of *V*_*i*_ can be rewritten as:

(3) $$\begin{array}{l} \begin{array}{c} \;V_{i}\left(t\right)=\underset{j\in \text{pre}}{\sum }W_{ij}P_{ij}\left(t\right),\\ \tau_{mem}\frac{\text{d}}{\text{d}t}P_{ij}\left(t\right)=-P_{ij}\left(t\right)+Q_{ij}\left(t\right),\\ \;\tau_{syn}\frac{\text{d}}{\text{d}t}Q_{ij}\left(t\right)=-Q_{ij}\left(t\right)+S_{j}\left(t\right). \end{array} \end{array}$$

The states *P* and *Q* describe the traces of the membrane and the current-based synapse, respectively. For each incoming spike, the trace *Q* undergoes a jump of height 1 and otherwise decays exponentially with a time constant τ_syn_. Weighting the trace *Q*_*ij*_ with the synaptic weight *W*_*ij*_ results in the PSPs of neuron *i* caused by input neuron *j*.

All efferent synapses with identical time constants have identical dynamics. By linearity of *P* and *Q*, the state of the synapse can be described by a single synaptic variable per pre-synaptic neuron (Brette et al., 2007). In the equation above, this is evident by the fact that *P*_*ij*_ and *Q*_*ij*_ are only driven by *S*_*j*_, and so the index *i* can be dropped. This results in as many *P* and *Q* variables as there are pre-synaptic neurons, independently of the number of synapses. This strategy is commonly used in synapse circuits in neuromorphic hardware to reduce circuit area (Bartolozzi and Indiveri, 2006), and in software simulations of spiking neurons to improve memory consumption and computation time (Brette et al., 2007).

#### Discrete Spike Response Model of the Neuron and Synapse Dynamics

Because a computer will be used to simulate the dynamics, the dynamics are simulated in discrete time. We denote the simulation time step with Δ*t*. We also make the layerwise organization of the network apparent with the superscript *l* denoting the layer to which the neuron belongs. The dynamical equations in Equations (1) and (3) are expressed in discrete time as:

(4) $$\begin{array}{l} \begin{array}{c} U_{i}^{l}\left[t\right]=\underset{j}{\sum }W_{ij}^{l}P_{j}^{l}\left[t\right]-\rho R_{i}^{l}\left[t\right]+b_{i}^{l},\\ \;S_{i}^{l}\left[t\right]=\Theta \left(U_{i}^{l}\left[t\right]\right), \end{array}\;\begin{array}{c} P_{j}^{l}\left[t+\Delta t\right]=\alpha P_{j}^{l}\left[t\right]+\left(1-\alpha \right)Q_{j}^{l}\left[t\right],\\ Q_{j}^{l}\left[t+\Delta t\right]=\beta Q_{j}^{l}\left[t\right]+\left(1-\beta \right)S_{j}^{l-1}\left[t\right],\\ R_{i}^{l}\left[t+\Delta t\right]=\gamma R_{i}^{l}\left[t\right]+\left(1-\gamma \right)S_{i}^{l}\left[t\right], \end{array} \end{array}$$

where the constants $\alpha =\exp\left(-\frac{\Delta t}{\tau_{\text{mem}}}\right)$, $\gamma =\exp\left(-\frac{\Delta t}{\tau_{\text{ref}}}\right)$, and $\beta =\exp\left(-\frac{\Delta t}{\tau_{\text{syn}}}\right)$ reflect the decay dynamics of the membrane potential *U*, the refractory state *R* and the synaptic state *Q* during a Δ*t* timestep. Note that Equation (4) is equivalent to a discrete-time version of the Spike Response Model (SRM_0_) with linear filters (Gerstner and Kistler, 2002).

### 2.2. Deep Learning With Local Losses

Loss functions are almost always defined using the network output at the top layer. Assuming a global cost function $L\left(S^{N}\right)$ defined on the spikes *S*^*N*^ of the top layer and targets Ŷ, the gradients with respect to the weights in layer *l* are:

(5) $$\begin{array}{l} \frac{\partial L\left(S^{N}\right)}{\partial W_{ij}^{l}}=\frac{\partial L\left(S^{N}\right)}{\partial S_{i}^{l}}\frac{\partial S_{i}^{l}}{\partial U_{i}^{l}}\frac{\partial U_{i}^{l}}{\partial W_{ij}^{l}}. \end{array}$$

The factor $\frac{\partial L\left(S^{N}\right)}{\partial S_{i}^{l}}$ captures the backpropagated errors, i.e., how changing the output of neuron *i* in layer *l* modifies the global loss. This problem is known as the credit assignment problem. It generally involves non-local terms, including the activity of other neurons, their errors, and their temporal history. Thus, using local information only, a neuron in a deep layer cannot infer how a change in its activity will affect the top-layer cost. An increasing body of work is showing that approximations to the backpropagated errors in SNNs can allow local learning, for example in feedback alignment (Lillicrap et al., 2014). However, maintaining the history of the dynamics efficiently remains a challenging and open problem. While it is possible to use BPTT methods to compute these errors, this comes at a significant cost in memory and computation (Williams and Zipser, 1989), and is not consistent with the constraint of local information.

We address this conundrum using deep local learning (Mostafa et al., 2017). We focus on a form of deep local learning that attaches random readouts to deep layers and defines auxiliary cost functions over the readout. These auxiliary cost functions provide a task-relevant source of error for neurons in deep layers. The random readout is obtained by multiplying the neural activations with a random and fixed matrix. Training deep layers using auxiliary local errors that minimize the cost locally still allows the network as a whole to reach a small top-layer cost. As explained in Mostafa et al. (2017), minimizing a local readout's classification loss puts pressure on deep layers to learn useful task-relevant features, which allow the random local classifiers to solve the task. Moreover, each layer builds on the features of the previous layer to learn features that are further disentangled with respect to the categories for its local random classifier compared to the previous layer. Thus, even though no error information propagates downwards through the layer stack, the layers indirectly learn useful hierarchical features that end up minimizing the cost at the top layer. Although the reasons for the effectiveness of local errors in deep network is intriguing and merits further work, it is orthogonal to the scope of this article. In this article, we focus on the fact that, provided local loss functions, surrogate learning in deep spiking neural networks becomes particularly efficient.

### 2.3. Deep Continuous Local Learning (DECOLLE)

As discussed above, in DECOLLE, we attach a random readout to each of the *N* layers of spiking neurons:

$$Y_{i}^{l}=\underset{j}{\sum }G_{ij}^{l}S_{j}^{l},$$

where $G_{ij}^{l}$ are fixed, random matrices (one for each layer *l*) and Θ is an activation function. The global loss function is then defined as the sum of the layerwise loss functions defined on the random readouts, *i.e*. $L=\overset{N}{\underset{l=1}{\sum }}L^{l}\left(Y^{l}\right)$. To enforce locality, DECOLLE sets to zero all non-local gradients, i.e., $\frac{\partial L^{l}}{\partial W_{ij}^{m}}=0$ if *m* ≠ *l*. With this assumption, the weight updates at each layer become:

(6) $$\begin{array}{l} \Delta W_{ij}^{l}=-\eta \frac{\partial L^{l}}{\partial W_{ij}^{l}}=-\eta \frac{\partial L^{l}}{\partial S_{i}^{l}}\frac{\partial S_{i}^{l}}{\partial W_{ij}^{l}}, \end{array}$$

where η is the learning rate. Assuming the loss function depends only on variables in same time step, the first gradient term on the right hand side, $\frac{\partial L^{l}}{\partial S_{i}^{l}}$, can be trivially computed using the chain rule of derivatives. Applying the chain of derivatives to the second gradient term yields:

$$\frac{\partial S_{i}^{l}}{\partial W_{ij}^{l}}=\frac{\partial \Theta \left(U_{i}^{l}\right)}{\partial U_{i}^{l}}\frac{\partial U_{i}^{l}}{\partial W_{ij}^{l}}.$$

Due to the sparse, binary activation of spiking neurons, this expression vanishes everywhere except at 0, where it is infinite (Neftci et al., 2019). To solve this problem, parameter updates in DECOLLE are based on a differentiable but slightly different version of the task-performing network. This approach was previously described as surrogate gradient-based learning (Zenke and Ganguli, 2017; Neftci et al., 2019):

$$\frac{\partial S_{i}^{l}}{\partial W_{ij}^{l}}=\sigma^{\prime }\left(U_{i}^{l}\right)\frac{\partial U_{i}^{l}}{\partial W_{ij}^{l}},$$

where $\sigma^{\prime }\left(U_{i}^{l}\right)$ is the surrogate gradient of the non-differentiable step function $\Theta \left(U_{i}^{l}\right)$. The rightmost term is computed as:

$$\frac{\partial U_{i}^{l}}{\partial W_{ij}^{l}}=P_{j}^{l}-\rho \frac{\partial R_{i}^{l}}{\partial W_{ij}^{l}}.$$

The terms involving $R_{i}^{l}$ are difficult to calculate because they depend on the spiking history of the neuron. As in Superspike, we ignore these dependencies and use regularization to favor low firing rates, a regime in which the $R_{i}^{l}$ has a negligible effect on the membrane dynamics. Putting all three terms together, we obtain the DECOLLE rule governing the synaptic weight update:

(7) $$\begin{array}{l} \Delta W_{ij}^{l}=-\eta \frac{\partial L^{l}}{\partial S_{i}^{l}}\sigma^{\prime }\left(U_{i}^{l}\right)P_{j}^{l}. \end{array}$$

In the special case of the Mean Square Error (MSE) loss for layer *l*, described as

$$L^{l}=\frac{1}{2}\underset{i}{\sum }{\left(Y_{i}^{l}-{Ŷ}_{i}^{l}\right)}^{2},$$

the DECOLLE rule becomes

(8) $$\begin{array}{l} \begin{array}{c} \Delta W_{ij}^{l}=-\eta \text{ erro}{\text{r}}_{i}^{l}\sigma^{\prime }\left(U_{i}^{l}\right)P_{j}^{l},\\ \text{erro}{\text{r}}_{i}^{l}=\underset{k}{\sum }G_{ki}^{l}\left(Y_{k}^{l}-{Ŷ}_{k}^{l}\right), \end{array} \end{array}$$

where Ŷ^*l*^ is the pseudo-target vector for layer *l*.

#### 2.3.1. Memory Complexity of DECOLLE

The variables *P* and *U* required for learning are local and readily available from the forward dynamics. Because the errors are computed locally to each layer, DECOLLE does not need to store any additional intermediate variables, i.e., there is no space requirement for the parameter update computation. The same neural traces *P* and *Q* maintained during the forward pass are sufficient (see section 2.4). The computational cost of the weight update is the same as the Widrow-Hoff rule (one addition and two products per connection, see Equation 8). This makes DECOLLE significantly cheaper to implement compared to BPTT for training SNN, e.g., SLAYER (Shrestha and Orchard, 2018) which scales spatially as *O*(*NT*), where *T* is the number of timesteps (see Appendix section 5.2 for details on scaling).

#### 2.3.2. Sign-Concordant Feedback Alignment in the Local Layers

The gradients of the local losses $L_{i}^{l}$ involve backpropagation through the local random projection *Y*^*l*^. This is a non-local operation as it requires the symmetric transpose of the matrix *G*. This raises a weight transport problem, whereby the synaptic weight must be “transported” from one neuron to another. In a von Neumann computer, this is not a problem since memory is shared across processes. However, if memory is local, then a dedicated process must transmit this information. Feedback alignment in non-spiking networks was demonstrated to overcome this problem at a cost in accuracy (Mostafa et al., 2017). In our experiments, we use sign-concordant feedback weights to compute the gradients of the local losses: the backward weights have the same sign as the forward ones, but subject to fixed multiplicative Gaussian noise. The noise here reflects the fact that weights do not need to be exactly symmetric. This assumption is the most plausible scenario in mixed-signal neuromorphic devices, where connections can be programmed with the same sign bidirectionally, but the effective weights are subject to fabrication mismatch (Neftci et al., 2011). Since the weights in the local readouts are fixed, there is no weight transport problem during learning. Thus, the computation of the errors can be carried out using another random matrix *H*^*l*^ (Lillicrap et al., 2016) whose elements are equal to $H_{ij}^{l}=G_{ij}^{l,T}\omega_{ij}^{l}$ with a Gaussian distributed $\omega_{ij}^{l}\sim N\left(1,\frac{1}{2}\right)$. To enforce sign-concordance, all values $\omega_{ij}^{l}$ below zero were set to zero.

#### 2.3.3. Biological Plausibility of DECOLLE and Suitability for Neuromorphic Hardware

Equation (8) consists of three factors, one modulatory (*error*_*i*_), one post-synaptic [$\sigma^{\prime }\left(U_{i}^{l}\right)$], and one pre-synaptic ($P_{j}^{l}$). These types of rules are often termed three-factor rules, which have been shown to be consistent with biology (Pfister et al., 2006), while being compatible with a wide number of unsupervised, supervised, and reinforcement learning paradigms (Urbanczik and Senn, 2014). The terms *P* and *Q* represent neural and synaptic states that are readily available at the neuron. In our previous work and general experience, the shape of the surrogate function σ does not play a major role in DECOLLE^1^. The surrogate function σ can be a piecewise linear function (Neftci et al., 2017), such that σ′ becomes a boxcar function. This corresponds to a learning update that is gated by the post-synaptic membrane potential, and is reminiscent of membrane voltage-based rules, where spike-driven plasticity is induced only when membrane voltage is inside an eligibility window (Brader et al., 2007; Chicca et al., 2013).

In the derivation of the DECOLLE rule, we used an instantaneous readout function *Y*^*l*^ in the sense that it did not depend on states of the previous time step. In biology, this readout would be carried out by spiking neurons. This introduces a temporal dependency. As in SuperSpike, this temporal dependency significantly increases the complexity of the learning, and is costly to implement in neuromorphic hardware. One solution is to compute the errors using spiking neurons with dynamics faster than those of the hidden neurons. In mixed signal hardware, this can be achieved through fast membrane and synaptic time constants. In digital hardware this could be achieved using a dedicated logic block.

#### 2.3.4. Regularization and Implementation Details

From a technological point of view, SNNs are interesting when the spike rate is low as dedicated neuromorphic hardware can directly exploit this sparsity to reduce computations by the same factor (Merolla et al., 2014; Davies et al., 2018). To ensure reasonable firing rates and prevent sustained firing, we use two regularizers. One keeps *U* below to the firing threshold on average, and one activity regularizer enforces a minimum firing rate in each layer. The final loss function is:

(9) $$\begin{array}{l} L_{g}=\underset{l}{\sum }L^{l}+\lambda_{1}{\langle {\left[U_{i}^{l}+0.01\right]}^{+}\rangle }_{i}+\lambda_{2}{\left[0.1-{\langle U_{i}^{l}\rangle }_{i}\right]}^{+} \end{array}$$

where 〈·〉_*i*_ denotes averaging over index *i*, [·]^+^ is a linear rectification, and λ_1_, λ_2_ are hyperparameters. The minimum firing rate regularization is included to prevent the layers becoming completely silent during the training. Our experiments used a piecewise linear surrogate activation function, such that its derivative becomes the boxcar function σ′(*x*) = 1 if *x* ∈ [−0.5, 0.5] and 0 otherwise.

In all our experiments, weight updates are made for each time step of the simulation. We use the AdaMax optimizer (Kingma and Ba, 2014) with parameters β_1_ = 0, β_2_ = 95 and learning rate 10^−9^, and a smooth L1 loss. Biases were used for all layers and trained in all DECOLLE layers. The weights *G*^*l*^ used for the local readouts were initialized uniformly. PyTorch code and a tutorial are publicly available on Github^2^. DECOLLE is simulated using mini-batches to leverage the GPU's parallelism.

### 2.4. Computational Graph and Implementation Using Automatic Differentiation

Perhaps one of the strongest advantages of DECOLLE is its out-of-the-box compatibility with Automatic Differentiation (AD) tools for implementing gradient BP. AD is a technology recently incorporated in machine learning frameworks to automatically compute gradients in a computational graph^3^. AD operates on the principle that all numerical computations are compositions of a finite set of elementary operations for which derivatives are known. By combining the derivatives of the operations through the chain rule of derivatives, the derivative of the overall composition can be computed in a single pass (Baydin et al., 2017).

In practice, machine learning frameworks augment each elementary computation with its corresponding derivative function. As the desired operation is constructed, the dependencies with other variables are recorded as a computational graph. To perform gradient BP, after a forward pass, a backward pass computes all the derivatives of the operations in the graph. The root node of the reverse graph is typically a scalar loss function, and the leaf nodes are generally inputs and parameters of the network. After the backward pass, the gradients of all leaf nodes are applied to the trained parameters or inputs according to the optimization routine (e.g., Adam or similar).

**Figure 2:**
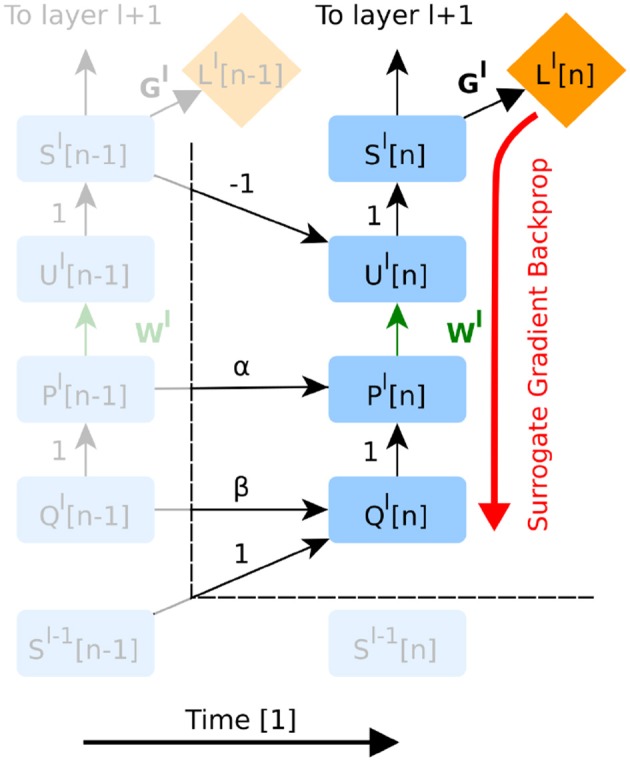
The unfolded computational graph of a feedforward SNN. Time flows to the right. Only temporal dependencies between timestep *n* − 1 and *n* are shown here. Green edges indicate variables trained in the presented version of DECOLLE. Red edges indicate the flow of the gradients. Note that this graph is similar to that of a simple recurrent neural network. The forward RTRL approach combined with local errors means that errors do not propagate through neurons and across layers, as all the information required for learning is available at the layer and the current time step *n*. For implementation purposes however, autodifferentiation can be used to compute gradients within the neuron and time step (see Appendix section 2.4 for details). To avoid clutter, the node for *R* has been omitted.

SNNs being a special case of recurrent neural networks, it is possible to apply AD to the full graph (Shrestha and Orchard, 2018). On the other hand, DECOLLE only requires backpropagating through a subgraph corresponding to one layer and within the same time step (Figure 2). This is because the information necessary for computing the gradients (*P*, *Q*, *R*, and *U*) is carried forward in time, and because local loss functions provide gradients for each layer.

AD in DECOLLE thus computes the gradients, locally, for each layer within each timestep. Because some operations in the subgraph can be non-differentiable (such as the spiking nonlinearity), we call this the “surrogate gradient backprop” (Figure 2). This integration allows leveraging the layers, operations, optimizers and cost functions provided by the software. All experiments under the Experiments section use AD to compute derivatives. To prevent AD from unnecessarily backpropagating in time, we rely on special “stop-gradient” operations. In the Appendix, we provide pseudocode and discussion of how this can be achieved.

## 3. Experiments

### 3.1. Regression With Poisson Spike Trains

To illustrate the inner workings of DECOLLE, we first demonstrate DECOLLE in a regression task. A three-layer fully connected network consisting of 512 neurons each is stimulated with a fixed 500 ms Poisson spike train. Each layer in the network is trained with a different pseudo-target: Ŷ^1^, a ramp function; Ŷ^2^, a high-frequency sinusoidal function and Ŷ^3^, a low-frequency sinusoidal function. Figure 1 illustrates the states of the neurons. For illustration purposes, the recording of the neural states was made in the absence of parameter updates (i.e., the learning rate is 0). The refractory mechanism decreases the membrane potential after the neuron spikes (*U*[*t*]). As discussed in the methods we use regularization on the membrane potential to keep the neurons from sustaining high firing rates and an activity regularizer to maintain a minimum firing rate. Updates to the weight are made at each time step and can be non-zero when the derivative of the activation function σ′(*U*) and *P* are non-zero. The magnitude and direction of the update are determined by the error. Note that, in effect, the error is randomized as a consequence of the random local readout. The network learned to use the input spike times to reliably produce the targets.

### 3.2. N-MNIST

The N-MNIST dataset was recorded with a Dynamic Vision Sensor (DVS) (Lichtsteiner et al., 2008) mounted on a pan-tilt unit performing microsaccadic motions in front of a screen displaying samples from the MNIST dataset (Orchard et al., 2015). Unlike standard imagers, the DVS records streams of events that signal the temporal intensity changes at each of its 128 × 128 pixels. For each of the 10 digits, we used 2,000 samples for training and 100 samples for testing. The samples are cropped spatially from 34 × 34 to 32 × 32 and temporally to 300 ms for both the train and test set. The network is simulated with a 1ms resolution—in other words, we sum up events in 1 ms time bins. No further pre-processing is applied to the events.

Events are separated in two channels with respect to their polarity. A N-MNIST sample is therefore represented as a tensor of shape 300 × 2 × 32 × 32, stacked into mini-batch of 500 samples. The DECOLLE network is fed with 1 ms slices of the input at a time. We relied on the same three-layer convolutional architecture used in the DvsGesture task described below. After a “burn-in” period of 50 ms during which no update is made, gradient updates are performed at every simulation step. Hence, there are 250 weight updates per mini-batch. While the relevance of the time domain in N-MNIST is debatable (Iyer et al., 2018), this experiment shows that the neural dynamics of our network leads to successful classifications in under 300 ms.

**Figure 3:**
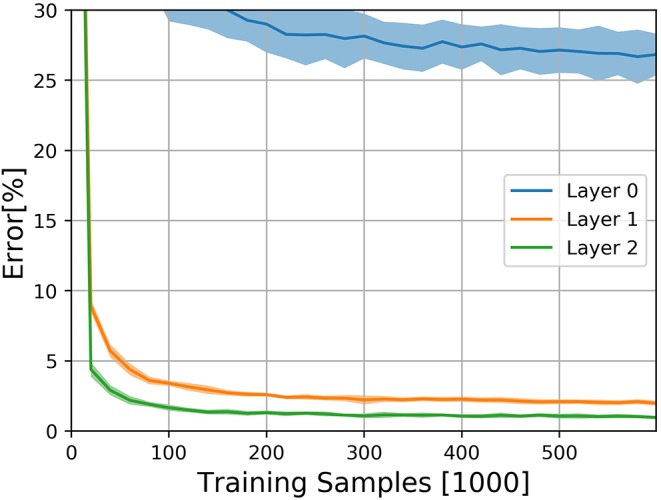
Classification results on the N-MNIST dataset for the three DECOLLE layers. Classification Error for the N-MNIST task during learning for all local errors associated with the convolutional layers. Shadings indicate standard deviation across the 10 runs.

The results on the N-MNIST dataset are shown in Figure 3. The experiment was performed 10 times with different random seeds. DECOLLE's final error is 0.96±0.12% for the third layer with 600,000 training iterations. We note that, due to the large memory requirements, it is not practical to train the DECOLLE convolutional network using BPTT. Hence we cannot provide BPTT baselines.

### 3.3. DvsGesture

**Figure 4:**
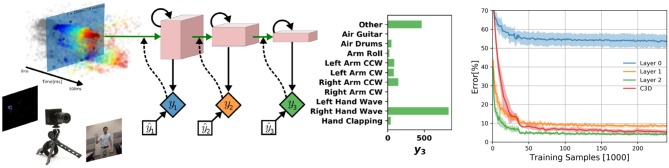
**(Left)** DECOLLE setup for DvsGesture recognition. Learning was performed on the dataset provided with Amir et al. (2017) and consists of 11 gestures. The network consisted of three convolutional layers with max-pooling. A local classifier is attached to every layer and followed by dropout for regularization. DECOLLE is fed with 1 ms integer frames. **(Right)** Classification Error for the DvsGesture task during learning for all local errors associated with the convolutional layers . Shadings indicate standard deviation across runs (5 runs for C3D, 10 runs for DECOLLE).

We test DECOLLE at the more challenging task of learning gestures recorded using a DVS. Amir et al. recorded the DvsGesture dataset using a DVS, which comprises 1,342 instances of a set of 11 hand and arm gestures, collected from 29 subjects under three different lighting conditions (Amir et al., 2017). The unique features of each gesture are embedded in the stream of events. The event streams were downsized from 128 × 128 to 32 × 32 (events from four neighboring pixels were summed together as a common stream) and binned in frames of 1ms, the effective time step of the GPU-based simulation (Figure 4). During training, a sample consisted of 500 ms-long slices of the sample. To maximize the use of the dataset, the starting point of the slice was picked randomly, but such that a full 500 ms sequence could be constructed. The sequences were presented to the network in mini-batches of 72 samples. Testing sequences were 1, 800 ms-long, each starting from the beginning of each recording (288 testing sequences). Note that since the shortest recording in the test set is 1, 800 ms, this duration was selected to simplify and speed up the evaluation. The classification is obtained by counting spikes at the output starting from a burn-in period of 50 ms and selecting as output class the neuron that spiked the most. Test results from the DECOLLE network are reported with the dropout layer kept active, as this provided better results. Contrary to Amir et al. (2017), we did not use stochastic decay and the neural network structure is a three-layer convolutional neural network, loosely adapted from Springenberg et al. (2014). We did not observe significant improvement by adding more than three convolutional layers. In shallow convolutional neural networks, it is common to use larger kernel sizes (LeCun et al., 1998; Kubilius et al., 2019) (Table 2). Since the input sizes were 32 × 32, we used 7 × 7 kernel sizes in DECOLLE to ensure that the receptive field of neurons in the last layer covered the input. The optimal hyperparameters were found by a combination of manual and grid search. The learning rate was divided by 5 every 500 steps.

We compared with C3D and energy-efficient deep networks (EEDN). EEDN is a convolutional deep neural network architecture that can be trained offline (e.g., on a GPU) and deployed on the IBM TrueNorth chip (Esser et al., 2016). EEDN was applied to DVS gestures and provides an important benchmark on this task (Amir et al., 2017). Because EEDN was not designed to utilize the temporal dynamics of the spiking neurons in IBM TrueNorth chip, time is represented using the channel dimension of 2D convolutional networks. This approach limits the length of the sequence that EEDN can process. To overcome this, Amir et al. (2017) used a sliding window filter. C3D is a 3D convolutional network commonly used for spatiotemporal classification in videos (Tran et al., 2015), where the dimensions are time, height, and width. Using 3D kernels, C3C can learn spatiotemporal patterns. The network was similar to Tran et al. (2015) except that is was adapted for 32 × 32 frames and using half of the features per layer (see Appendix for network layers). We note that the C3D network is deeper and wider than the DECOLLE network. We found that 16 × 32 × 32 frames, where each of the 16 representing 32ms slices of the DVS data performed best.

Overall, DECOLLE's performance is comparable or better than other published SNN implementations that use BP for training (Table 1, Figure 4) and close to much larger C3D networks. DECOLLE reached the reported accuracies after two orders of magnitude fewer iterations and smaller network compared to the IBM EEDN case (Table 1) (Amir et al., 2017).

**Table 1:** Classification error at the DvsGesture task.

**Model**	**Error**	**Training**	**Iterations**	**References**
DECOLLE	**4.46 ± 0.16%**	**Online**	**0.16M**	This Work
SLAYER	6.36 ± 0.49 %	BPTT	0.27M	Shrestha and Orchard, 2018
C3D	5.46 ± 1.06%	BPTT	0.32M	Tran et al., 2015
IBM EEDN	8.23% (5.51%)	BPTT	64M	Amir et al., 2017

*The IBM EEDN error in parentheses refers to the case with sliding window filter. Bold values emphasize that this work achieves the lowest error*.

**Table 2:** DECOLLE Neural network used for the DvsGesture dataset.

**Layer type**	**#**	**Data type**	**Dimensions**
DVS	2	AEDAT 3.1	128 × 128
Downsample (Sum)	2	Integer	32 × 32
7 × 7 Conv	64	Float	30 × 30
2 × 2 MaxPool	64	Float	15 × 15
Spiking Non-linearity		Binary	
Dropout (*p* = 0.5)		Float	
Dense	11	Float	11
7 × 7 Conv	128	Float	13 × 13
Spiking Non-linearity		Binary	
Dropout (*p* = 0.5)		Binary	
Dense	11	Float	11
7 × 7 Conv	128	Float	11 × 11
2 × 2 MaxPool	128	Float	5 × 5
Spiking Non-linearity		Binary	
Dropout (*p* = 0.5)		Binary	
Dense	11	Float	11

*Note that dense layers are used for the local classifiers only and were not fed to the subsequent convolutional layers. AEDAT 3.1 is a data format used for event-based data. The spiking nonlinearity was always applied after the pooling layers. Dropout layers were left active during testing*.

**Figure 5:**
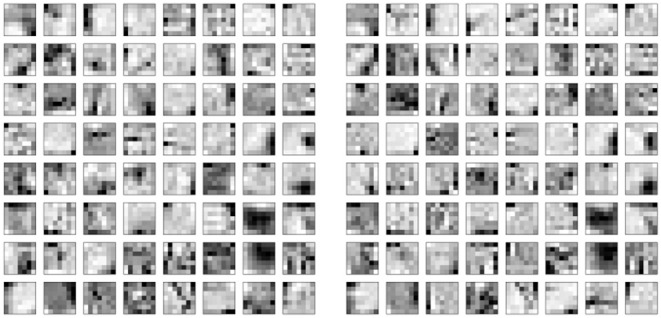
7 × 7 Filters learned in the positive polarity channel **(Left)** and negative polarity channel **(Right)** of the first convolutional layer. The similarity of the kernels across the two polarities reflects the DVS data, where leading edges and trailing edges co-occur with opposite polarities.

Interestingly, the first layer of DECOLLE has a low classification accuracy. A similar effect is observed in non-spiking neural networks Mostafa et al. (2017). The local classifier errors improve for the second and third hidden layers compared to the first hidden layer. This is an indication that the network is able to make use of depth to obtain better accuracy. An examination of the filters learning in the first convolutional layer shows filters of varying frequencies and orientations (Figure 5). Interstingly, the filters on the positive and negative channels of the DVS are similar, but exhibit small variations that are consistent with motion. This correlation is consistent with the DVS data, where leading edges of one polarity co-occur with trailing edges of opposite polarity.

## 4. Conclusion

Understanding and deriving neural and synaptic plasticity rules that can enable hidden weights to learn is an ongoing quest in neuroscience and neuromorphic engineering. From a machine learning perspective, locality, and differentiability are key issues of the spiking neuron model operations. While the latter problem is now being tackled with surrogate gradient approaches, how to achieve this in deep networks in a scalable and local fashion is still an open question.

We presented a novel synaptic plasticity rule, DECOLLE, derived from a surrogate gradient approach with linear scaling in the number of neurons. The rule draws on recent work in surrogate gradient descent in spiking neurons and local learning with layerwise classifiers. The linear scalability is obtained through a (instantaneous) rate-based cost function on the local classifier. The simplicity of the DECOLLE rule equation makes it amenable for direct exploitation of existing machine learning software libraries. Thanks to the surrogate gradient approach, the updates computed through automatic differentiation are equal to the DECOLLE update. This enables the leveraging of a wide variety of machine learning frameworks for implementing online learning of SNNs.

Updates in DECOLLE are performed at every time step, in accordance with the continuity of the leaky I&F dynamics. This can lead to a large number of updates and inefficient implementations in hardware. To tackle this problem, updates can be made in an error-triggered fashion, as discussed in Payvand et al. (2020). A direct consequence of the local classifiers is the lack of cross-layer adaptation of the layers. To tackle this problem, one could use meta-learning to adapt the random matrix in the classifier. In effect, the meta-learning loop would act as the outer loop in the synthetic gradients approach Jaderberg et al. (2016). The notion that a “layer” of neurons specialized in solving certain problems and sensory modalities is natural in computational neurosciences and can open multiple investigation avenues for understanding learning and plasticity in the brain.

DECOLLE is a departure from standard SNNs trained with Hebbian spike-timing-dependent plasticity, as it uses a normative learning rule that is partially derived from first principles. Models of this type can make use of standard processors where it makes the most sense (i.e., readout, cost functions etc.) and neuromorphic dedicated hardware for the rest. Because it leverages the best of both worlds, DECOLLE is poised to make SNNs take off in event-based computer vision.

## Data Availability Statement

The raw data supporting the conclusions of this article will be made available by the authors, without undue reservation, to any qualified researcher.

## Author Contributions

JK, HM, and EN wrote the paper and conceived the experiments. JK and EN ran the experiments and analyzed the data.

## Conflict of Interest

The authors declare that the research was conducted in the absence of any commercial or financial relationships that could be construed as a potential conflict of interest.

## Footnotes

^1^Conversely, the particular surrogate function is reported to play an important role in BPTT (Bellec et al., 2018). This is likely due the product of the gradient approximations carried across multiple layers. This in turn can cause vanishing or exploding gradients.

^2^https://github.com/nmi-lab/decolle-public

^3^Gradient BP is a special case of reverse mode AD, see Baydin et al. (2017) for complete review.

## References

[B1] Amir A. Taba B. Berg D. Melano T. McKinstry J. Di Nolfo C. (2017). “A low power, fully event-based gesture recognition system,” in Proceedings of the IEEE Conference on Computer Vision and Pattern Recognition (Honolulu, HI), 7243–7252. 10.1109/CVPR.2017.781

[B2] Baldi P. Sadowski P. Lu Z. (2017). Learning in the machine: the symmetries of the deep learning channel. Neural Netw. 95, 110–133. 10.1016/j.neunet.2017.08.008 28938130

[B3] Bartolozzi C. Indiveri G. (2006). “Silicon synaptic homeostasis,” in Brain Inspired Cognitive Systems, BICS 2006 (Lesvos), 1–4.

[B4] Bartunov S. Santoro A. Richards B. Marris L. Hinton G. E. Lillicrap T. (2018). “Assessing the scalability of biologically-motivated deep learning algorithms and architectures,” in Advances in Neural Information Processing Systems (Montréal, QC), 9368–9378.

[B5] Baydin A. G. Pearlmutter B. A. Radul A. A. Siskind J. M. (2017). Automatic differentiation in machine learning: a survey. J. Mach. Learn. Res. 18, 5595–5637.

[B6] Bellec G. Salaj D. Subramoney A. Legenstein R. Maass W. (2018). Long short-term memory and learning-to-learn in networks of spiking neurons. arXiv [Preprint]. arXiv:1803.09574.

[B7] Bellec G. Scherr F. Hajek E. Salaj D. Legenstein R. Maass W. (2019). Biologically inspired alternatives to backpropagation through time for learning in recurrent neural nets. arXiv [Preprint]. arXiv:1901.09049.

[B8] Bohte S. M. Kok J. N. La Poutré J. A. (2000). “Spikeprop: backpropagation for networks of spiking neurons,” in ESANN (Bruges), 419–424.

[B9] Brader J. Senn W. Fusi S. (2007). Learning real world stimuli in a neural network with spike-driven synaptic dynamics. Neural Comput. 19, 2881–2912. 10.1162/neco.2007.19.11.2881 17883345

[B10] Brette R. Rudolph M. Carnevale T. Hines M. Beeman D. Bower J.. (2007). Simulation of networks of spiking neurons: a review of tools and strategies. J. Comput. Neurosci. 23, 349–398. 10.1007/s10827-007-0038-6 17629781 PMC2638500

[B11] Chicca E. Stefanini F. Indiveri G. (2013). Neuromorphic electronic circuits for building autonomous cognitive systems. Proc. IEEE 102, 1367–1388. 10.1109/JPROC.2014.2313954

[B12] Clopath C. Büsing L. Vasilaki E. Gerstner W. (2010). Connectivity reflects coding: a model of voltage-based STDP with homeostasis. Nat. Neurosci. 13, 344–352. 10.1038/nn.2479 20098420

[B13] Courbariaux M. Hubara I. Soudry D. El-Yaniv R. Bengio Y. (2016). Binarized neural networks: Training deep neural networks with weights and activations constrained to+ 1 or-1. arXiv [Preprint]. arXiv:1602.02830.

[B14] Davies M. Srinivasa N. Lin T. H. Chinya G. Joshi P. Lines A. (2018). Loihi: a neuromorphic manycore processor with on-chip learning. IEEE Micro 38, 82–99. 10.1109/MM.2018.112130359

[B15] Eliasmith C. Anderson C. (2004). Neural Engineering: Computation, Representation, and Dynamics in Neurobiological Systems. MIT Press.

[B16] Esser S. K. Merolla P. A. Arthur J. V. Cassidy A. S. Appuswamy R. Andreopoulos A.. (2016). Convolutional networks for fast, energy-efficient neuromorphic computing. Proc. Natl. Acad. Sci. U.S.A. 113, 11441–11446. 10.1073/pnas.1604850113 27651489 PMC5068316

[B17] Gerstner W. Kistler W. (2002). Spiking Neuron Models. Single Neurons, Populations, Plasticity. Cambridge University Press. 10.1017/CBO9780511815706

[B18] Gerstner W. Kistler W. M. Naud R. Paninski L. (2014). Neuronal Dynamics: From Single Neurons to Networks and Models of Cognition. Cambridge University Press. 10.1017/CBO9781107447615

[B19] Gütig R. Sompolinsky H. (2006). The tempotron: a neuron that learns spike timing-based decisions. Nat. Neurosci. 9, 420–428. 10.1038/nn1643 16474393

[B20] Huayaney F. L. M. Nease S. Chicca E. (2016). Learning in silicon beyond STDP: a neuromorphic implementation of multi-factor synaptic plasticity with calcium-based dynamics. IEEE Trans. Circuits Syst. I 63, 2189–2199. 10.1109/TCSI.2016.2616169

[B21] Huh D. Sejnowski T. J. (2017). Gradient descent for spiking neural networks. arXiv [Preprint]. arXiv:1706.04698.

[B22] Iyer L. R. Chua Y. Li H. (2018). Is neuromorphic mnist neuromorphic? Analyzing the discriminative power of neuromorphic datasets in the time domain. arXiv [Preprint]. arXiv:1807.01013. 10.3389/fnins.2021.608567 PMC8027306 33841072

[B23] Jaderberg M. Czarnecki W. M. Osindero S. Vinyals O. Graves A. Kavukcuoglu K. (2016). Decoupled neural interfaces using synthetic gradients. arXiv [Preprint]. arXiv:1608.05343.

[B24] Jaeger H. (2001). The “echo state” approach to analysing and training recurrent neural networks-with an erratum note. Technical Report, German National Research Center for Information Technology GMD, Bonn.

[B25] Kaiser J. Mostafa H. Neftci E. (2018). Synaptic plasticity for deep continuous local learning. arXiv [Preprint]. arXiv:1812.10766. 10.3389/fnins.2020.00424 PMC7235446 32477050

[B26] Kingma D. P. Ba J. (2014). Adam: A method for stochastic optimization. arXiv Preprint. arXiv:1412.6980.

[B27] Kubilius J. Schrimpf M. Hong H. Majaj N. J. Rajalingham R. Issa E. B. (2019). Brain-like object recognition with high-performing shallow recurrent ANNs. arXiv [Preprint]. arXiv:1909.06161.

[B28] LeCun Y. Bottou L. Bengio Y. Haffner P. (1998). Gradient-based learning applied to document recognition. Proc. IEEE 86, 2278–2324. 10.1109/5.726791

[B29] Lee J. H. Delbruck T. Pfeiffer M. (2016). Training deep spiking neural networks using backpropagation. Front. Neurosci. 10:508. 10.3389/fnins.2016.00508 27877107 PMC5099523

[B30] Lichtsteiner P. Posch C. Delbruck T. (2008). An 128x128 120dB 15μs-latency temporal contrast vision sensor. IEEE J. Solid State Circuits 43, 566–576. 10.1109/JSSC.2007.914337

[B31] Lillicrap T. P. Cownden D. Tweed D. B. Akerman C. J. (2014). Random feedback weights support learning in deep neural networks. arXiv [Preprint]. arXiv:1411.0247.

[B32] Lillicrap T. P. Cownden D. Tweed D. B. Akerman C. J. (2016). Random synaptic feedback weights support error backpropagation for deep learning. Nat. Commun. 7:13276. 10.1038/ncomms13276 27824044 PMC5105169

[B33] Maass W. Natschläger T. Markram H. (2002). Real-time computing without stable states: a new framework for neural computation based on perturbations. Neural Comput. 14, 2531–2560. 10.1162/089976602760407955 12433288

[B34] Merolla P. A. Arthur J. V. Alvarez-Icaza R. Cassidy A. S. Sawada J. Akopyan F.. (2014). A million spiking-neuron integrated circuit with a scalable communication network and interface. Science 345, 668–673. 10.1126/science.1254642 25104385

[B35] Mostafa H. Ramesh V. Cauwenberghs G. (2017). Deep supervised learning using local errors. arXiv [Preprint]. arXiv:1711.06756. 10.3389/fnins.2018.00608 30233295 PMC6127296

[B36] Neftci E. Augustine C. Paul S. Detorakis G. (2017). Event-driven random back-propagation: Enabling neuromorphic deep learning machines. Front. Neurosci. 11:324. 10.3389/fnins.2017.00324 28680387 PMC5478701

[B37] Neftci E. Chicca E. Indiveri G. Douglas R. (2011). A systematic method for configuring VLSI networks of spiking neurons. Neural Comput. 23, 2457–2497. 10.1162/NECO_a_00182 21732859

[B38] Neftci E. O. Mostafa H. Zenke F. (2019). Surrogate gradient learning in spiking neural networks: Bringing the power of gradient-based optimization to spiking neural networks. IEEE Signal Process. Mag. 36, 51–63. 10.1109/MSP.2019.2931595

[B39] Orchard G. Jayawant A. Cohen G. K. Thakor N. (2015). Converting static image datasets to spiking neuromorphic datasets using saccades. Front. Neurosci. 9:437. 10.3389/fnins.2015.00437 26635513 PMC4644806

[B40] Payvand M. Fouda M. Eltawil A. Kurdahi F. Neftci E. (2020). “Error-triggered three-factor learning dynamics for crossbar arrays,” in 2020 IEEE International Conference on Artificial Intelligence Circuits and Systems (AICAS) (Genova). 10.1109/AICAS48895.2020.9073998

[B41] Pfister J.-P. Toyoizumi T. Barber D. Gerstner W. (2006). Optimal spike-timing-dependent plasticity for precise action potential firing in supervised learning. Neural Comput. 18, 1318–1348. 10.1162/neco.2006.18.6.1318 16764506

[B42] Rastegari M. Ordonez V. Redmon J. Farhadi A. (2016). “Xnor-net: Imagenet classification using binary convolutional neural networks,” in European Conference on Computer Vision (Amsterdam: Springer), 525–542. 10.1007/978-3-319-46493-0_32

[B43] Shrestha S. B. Orchard G. (2018). “Slayer: Spike layer error reassignment in time,” in Advances in Neural Information Processing Systems (Montréal, QC), 1412–1421.

[B44] Springenberg J. T. Dosovitskiy A. Brox T. Riedmiller M. (2014). Striving for simplicity: the all convolutional net. arXiv [Preprint]. arXiv:1412.6806.

[B45] Sussillo D. Abbott L. F. (2009). Generating coherent patterns of activity from chaotic neural networks. Neuron 63, 544–557. 10.1016/j.neuron.2009.07.018 19709635 PMC2756108

[B46] Tran D. Bourdev L. Fergus R. Torresani L. Paluri M. (2015). “Learning spatiotemporal features with 3D convolutional networks,” in Proceedings of the IEEE International Conference on Computer Vision (Santiago), 4489–4497. 10.1109/ICCV.2015.510

[B47] Urbanczik R. Senn W. (2014). Learning by the dendritic prediction of somatic spiking. Neuron 81, 521–528. 10.1016/j.neuron.2013.11.030 24507189

[B48] Williams R. J. Zipser D. (1989). A learning algorithm for continually running fully recurrent neural networks. Neural Comput. 1, 270–280. 10.1162/neco.1989.1.2.270

[B49] Zenke F. Ganguli S. (2017). Superspike: Supervised learning in multi-layer spiking neural networks. arXiv [Preprint]. arXiv:1705.11146. 10.1162/neco_a_01086 PMC6118408 29652587

