# Supplementary material for: Synaptic Plasticity Dynamics for Deep Continuous Local Learning (DECOLLE)
Source: Front Neurosci. 2020 May 12;14:424. doi: 10.3389/fnins.2020.00424 (PMC7235446; doi:10.3389/fnins.2020.00424)
Supplement: Supplementary file 1 [file Presentation_1.pdf]

Supplementary Information: Synaptic Plasticity Dynamics for Deep Continuous Local Learning (DECOLLE)

Jacques Kaiser,
FZI Research Center For Information Technology
Karlsruhe, Germany

Hesham Mostafa,
Department of Bioengineering
University of California San Diego
La Jolla, USA

Emre Neftci,
Department of Cognitive Sciences
Department of Computer Science
University of California Irvine, Irvine, USA

April 28, 2020

1 Implementation of DECOLLE using Autodifferentiation

The equations of Deep Continuous Local Learning (DECOLLE) are very similar to that of a simple recurrent neural network. However, rather than performing backpropagation through-time, the derivatives of U_i are computed by propagating the traces P_i forward in time as follows:

---

for $n = 0...T$ **do**
 {Advance State}
 for $l < 1...L$ **do**
  $U_i^l = \sum_j W_{ij}^l P_j^l - \rho R_i^l$
  $S_i = STOPGRAD(\Theta(U_i) - \sigma(U_i)) + \sigma(U_i)$
  if Sign-concordant feedback matrix **then**
   $Y_i = STOPGRAD(\sum_j G_{ij} S_j - \sum_j H_{ij} S_j) + \sum_j H_{ij} S_j$
  else
   $Y_i = \sum_j G_{ij} S_j$
  end if
  $L^l = f_{loss}(Y_k^l, \hat{Y}_k^l)$
  $W_{ij}^l = W_{ij}^l + \eta GRAD(L^l, W_{ij}^l)$
  $P_i^l = \alpha P_i^l + (1 - \alpha_i) Q_i^l$
  $Q_i^l = \beta Q_i^l + (1 - \beta_i) STOPGRAD(S_i^{l-1})$
  $R_i^l = \gamma R_i^l + (1 - \gamma_i) STOPGRAD(S_i^{l-1})$
 end for
end for

---

where f_{loss} is the loss function, *e.g.* MSE loss. STOPGRAD prevents the flow of gradients by setting them to zero. STOPGRAD(A-B)+B as above is a common construct used to compute gradients using a separate subgraph. In this

case, it is used to implement the surrogate gradient. Note that the time variable does not appear in the variables. This underlines that DECOLLE computation does not need to store the state histories, and that the variables necessary for computing the gradient (GRAD) are available within the same step. In our implementation, the weights are updated online, at every time step. The cost of making the parameter update is no more than accumulating the gradients at each time step, since parameter memory and dynamical state memory are the same. Therefore, there is no overhead in updating online. Note that this may not be the case in dedicated neuromorphic hardware or AI accelerators that require different memory structures for storing parameter memory.

2 Complexity Overhead for Various Spiking Neuron Gradient-Based Training Approaches

We provide additional detail on the complexity of DECOLLE compared to other learning methods. In the current implementation of DECOLLE, all weight updates are applied immediately, thus no additional memory is necessary to accumulate the gradients. In all other methods presented in (Tab. 1), the weight updates are applied in an epoch-wise fashion, which requires an additional variable to store the accumulated weights. However, this is an implementation choice which could have been made for methods other than DECOLLE as well. For this reason, the overhead of accumulating gradients in epoch-wise learning is ignored in the following calculations.

DECOLLE The state of P and Q must be maintained. These states are readily available from the forward pass, and therefore do not need to be stored specifically for learning. Space complexity is therefore $O(1)$. Each weight update requires MN_r multiplications to obtain M local errors. Each of these are multiplied by the number of inputs pN, resulting in $O(pNM + MN_r)$ time complexity, where p is the faction of connected neurons. Similarly to [2], the random weights in G^l can be computed using a random number generator, which requires one seed value per layer.

Superspike When using the Van Rossum Distance (VRD), the Superspike learning rule requires one trace per connection, resulting in a space complexity of $O(pNM)$. The additional complexity here compared to DECOLLE is caused by the additional filter in the Van Rossum distance. Note that if the learning is applied directly to membrane potentials, the space and time complexity is similar to that of DECOLLE.

eProp In the case when no future errors are used, the complexity of e-Prop [1] is similar to that of SuperSpike.

RTRL and BPTT The complexity of these techniques are discussed in detail in [3].

Method	Space	Time
DECOLLE	$O(1)$	$O(MN_r + pNM)$
SuperSpike (VRD)	$O(pNM)$	$O(pNM)$
e-Prop 1	$O(pNM)$	$O(pNM)$
RTRL	$O(pNM^2)$	$O(p^2N^2M^2)$
BPTT	$O(NT)$	$O(pNMT)$

Table 1: Complexity analysis of the gradient computation. N: Input neurons, M: Neurons in Layer, T: Length of Backpropagated Sequence, N_r: number of readout neurons in DECOLLE, p: ratio of connected neurons/total possible connections.

3 C3D Network

We used a standard 3D convolution network (C3D) for comparison with DECOLLE. In C3D, the temporal dimension is taken into account in the third dimension of the 3D convolution.

Layer Type	#	Data Type	Dimensions
DVS	2	AEDAT 3.1	128×128
Downsample (Sum) and Frame	2	Binary	$16 \times 32 \times 32$
3×3 Conv ReLU	32	Binary	$16 \times 32 \times 32$
$1 \times 2 \times 2$ MaxPool	32	Binary	$16 \times 16 \times 16$
3×3 Conv ReLU	64	Binary	$16 \times 16 \times 16$
$2 \times 2 \times 2$ MaxPool	64	Binary	$8 \times 8 \times 8$
3×3 Conv ReLU	128	Binary	$8 \times 8 \times 8$
3×3 Conv ReLU	128	Binary	$8 \times 8 \times 8$
$2 \times 2 \times 2$ MaxPool	128	Binary	$4 \times 4 \times 4$
3×3 Conv ReLU	256	Binary	$4 \times 4 \times 4$
3×3 Conv ReLU	256	Binary	$4 \times 4 \times 4$
$2 \times 2 \times 2$ MaxPool	256	Binary	$2 \times 2 \times 2$
3×3 Conv ReLU	256	Binary	$2 \times 2 \times 2$
3×3 Conv ReLU	256	Binary	$2 \times 2 \times 2$
$2 \times 2 \times 2$ MaxPool	256	Binary	$1 \times 2 \times 2$
Dense ReLU	1024	Float	1024
Dense ReLU	512	Float	512
Dense Softmax	11	Float	11

References

[1] Guillaume Bellec, Franz Scherr, Elias Hajek, Darjan Salaj, Robert Legenstein, and Wolfgang Maass. Biologically inspired alternatives to backpropagation through time for learning in recurrent neural nets. *arXiv preprint arXiv:1901.09049*, 2019.

[2] Hesham Mostafa, Vishwajith Ramesh, and Gert Cauwenberghs. Deep supervised learning using local errors. *arXiv preprint arXiv:1711.06756*, 2017.

[3] Ronald J Williams and David Zipser. Gradient-based learning algorithms for recurrent networks and their computational complexity. *Backpropagation: Theory, architectures, and applications*, 433, 1995.
